# Facilitating advance care planning in the general practice setting for patients with a chronic, life-limiting illness: protocol for a phase-III cluster-randomized controlled trial and process evaluation of the ACP-GP intervention

**DOI:** 10.1186/s12904-021-00796-1

**Published:** 2021-06-25

**Authors:** Julie Stevens, Peter Pype, Kim Eecloo, Luc Deliens, Koen Pardon, Aline De Vleminck

**Affiliations:** 1 Vrije Universiteit Brussel (VUB) & Ghent University, End-of-life Care Research Group, Laarbeeklaan 103, 1090 Brussels, Belgium; 2 Department of Family Medicine and Chronic Care, Vrije Universiteit Brussel, Brussels, Belgium; 3 Department of Public Health and Primary Care, Ghent University, Ghent, Belgium

**Keywords:** Advance care planning, Communication, General practice, Phase III, Randomized controlled trial, Complex intervention, Process evaluation

## Abstract

**Background:**

Advance care planning (ACP), a process of communication about patients’ preferences for future medical care, should be initiated in a timely manner. Ideally situated for this initiation is the general practitioner (GP). The intervention to improve the initiation of ACP for patients with a chronic life-limiting illness in general practice (ACP-GP) includes an ACP workbook for patients, ACP communication training for GPs, planned ACP conversations, and documentation of ACP conversation outcomes in a structured template. We present the study protocol of a Phase-III randomized controlled trial (RCT) of ACP-GP that aims to evaluate its effects on outcomes at the GP, patient, and surrogate decision maker (SDM) levels; and to assess the implementation process of the intervention.

**Methods:**

This RCT will take place in Flanders, Belgium. Thirty-six GPs, 108 patients with a chronic, life-limiting illness, and their (potential) SDM will be recruited, then cluster-randomized to the ACP-GP intervention or the control condition. The primary outcome for GPs is ACP self-efficacy; primary outcome for patients is level of ACP engagement. Secondary outcomes for GPs are ACP practices, knowledge and attitudes; and documentation of ACP discussion outcomes. Secondary outcomes for patients are quality of life; anxiety; depression; appointment of an SDM; completion of new ACP documents; thinking about ACP; and communication with the GP. The secondary outcome for the SDM is level of engagement with ACP. A process evaluation will assess the recruitment and implementation of the intervention using the RE-AIM framework.

**Discussion:**

While the general practice setting holds promise for timely initiation of ACP, there is a lack of randomized trial studies evaluating the effectiveness of ACP interventions implemented in this setting. After this Phase-III RCT, we will be able to present valuable evidence of the effects of this ACP-GP intervention, with the potential for offering a well-tested and evaluated program to be implemented in general practice. The results of the process evaluation will provide insight into what contributes to or detracts from implementation success, as well as how the intervention can be adapted to specific contexts or needs.

**Trial registration:**

Prospectively registered at with ISRCTN (ISRCTN12995230); registered 19/06/2020.

**Supplementary Information:**

The online version contains supplementary material available at 10.1186/s12904-021-00796-1.

## Background

Advance Care Planning (ACP) refers to “a process that supports adults at any age or stage of health in understanding and sharing their personal values, life goals, and preferences regarding future medical care” [1]. This may include the completion of a living will or Advance Directive (AD), which document wishes for future care should patients be unable to make their wishes known due to declining health or incapacity; and/or the appointing of a surrogate decision-maker (SDM), who can make care decisions in the place of the patient if the patient is unable to speak for themselves. ACP can lead to greater concordance between care preferences and care received [2], improved communication about the end of life with care providers [3], greater satisfaction with physician visits [4], and improved quality of end-of-life care [5]. It is a prerequisite for a good coordination of care, including palliative and end-of-life care, by making clear which medical decisions will be considered appropriate when the patient is unable to make such a decision themselves [6].

For patients with chronic, life-limiting illness (es), which are often marked by trajectories of steady illness progression or gradual health decline punctuated by acute deterioration [7], it is important that ACP is initiated in a timely manner so that sufficient time can be dedicated to conversations about values, goals and preferences [8]. ACP is intended as a continuous process of communication. For patients, engaging in ACP is not an isolated occurrence [9], but a complex behavior where readiness to engage in discrete behavior is an important precursor to action [10]. Especially suited to initiating these interactive discussions over multiple visits is the general practice setting. In Belgium, as in many other European countries, general practitioners (GPs) observe the patient’s health over the course of regular visits, often have a trusting relationship with the patient, and often are aware of the patient’s medical and social context [11, 12]. However, while the role of the GP in initiating ACP conversations is highlighted in guidelines of care [13], currently the process of ACP between patients and GP is not often initiated [14].

There is a lack of adequate practice models of initiation and implementation of ACP in general practice, and randomized trial studies evaluating the effectiveness of ACP interventions implemented in this setting are still largely absent. In light of this, a complex intervention for general practice has been developed [15, 16], and had subsequently been pilot-tested. The intervention was found to be feasible and acceptable. Based on the results of the pilot test, the intervention was adapted and is now being tested in a Phase-III trial [17]. This manuscript presents the research protocol for the Phase-III randomized controlled trial (RCT) study of the intervention. The Standard Protocol Items: Recommendations for Interventional Trials (SPIRIT) statement was applied to describe all relevant aspects of the trial (see Additional file 1) [18, 19].

### Objectives

The aim of this study is: To evaluate the effectiveness and mechanisms of action of a complex, multi-component ACP intervention, called ACP-GP, for patients with chronic, life-limiting illness (es), in the general practice setting, aimed at improving the readiness of patients to engage with ACP. The intervention will be compared to care as usual. Study objectives are:

**Table:** 

(1) To **test** the effectiveness of the ACP-GP intervention on: • the patient’s level of engagement with ACP (primary outcome at patient level) • the GP’s self-efficacy for conducting ACP (primary outcome at GP level) (2) To **explore** the effect of the ACP-GP intervention on: • patient quality of life; symptoms of anxiety; symptoms of depression; the appointment of a substitute decision-maker; completion of new ACP documents; thinking about ACP, and communication with the GP (secondary outcomes at patient level) • GP ACP practices, attitudes and knowledge about ACP, and the documentation of ACP discussions in the patient medical file (secondary outcomes at GP level) • the SDM’s level of engagement with ACP (secondary outcome at the SDM level) (3) To **evaluate** the recruitment and implementation process of the intervention in terms of its reach, efficacy, adoption, implementation, and maintenance; as reported by patients, their SDM if present, and GPs	

### Trial design

This study is a 2-arm cluster-RCT with a parallel group design, which compares the ACP-GP intervention (arm 1) to usual care (arm 2) of patients with a chronic life-limiting illness. It is a superiority trial which aims to establish whether the intervention is superior to usual care in its effectiveness. GPs, their patients, and the (potential) SDM of each patient will be recruited for participation. Randomization occurs at the level of the GP, with patients and their SDM clustered per GP. To determine effectiveness, outcomes will be measured at baseline (T0), during a first follow-up at 3 months (T1) and again at 6 months post-baseline (T2).

A process evaluation will be used to evaluate how the intervention was implemented and to understand which factors contributed to the results of the trial. This process evaluation follows the RE-AIM framework, which highlights essential factors to improving the adoption and implementation of evidence-based interventions: Reach, Efficacy, Adoption, Implementation, and Maintenance [20]. The process evaluation will span the duration of the intervention, as well as pre- and post-intervention evaluation.

## Methods

### Study setting

The intervention will be conducted in the setting of general practices the region of Flanders, Belgium.

### Eligibility criteria

Dutch-speaking GPs working in Flanders and Brussels, Belgium, are eligible to participate. GPs may practice in a group or solo setting, in urban, semi-urban, or rural areas. To reduce contamination risk, one GP per practice will be included. In order to participate, GPs also must be able to identify and include at least 3 eligible patients.

Eligible patients are those with a chronic, life-limiting illness (using indicators described in Table 1) for whom the GP answers “no” to the “surprise question”: “Would I be surprised if this patient were to die within the next 12 to 24 months?” [21]. This one-item screening tool assists in identifying patients with chronic, life-limiting illness who may benefit from the start of an ACP process [22, 23]. Patients for whom the GP would not be surprised if they were to die within the next 6 months will be excluded as the intervention will be tested over a period of 6 months.

**Table 1:** Inclusion and exclusion criteria for patients

Patient inclusion criteria	Patient exclusion criteria
Adults (> 18 years old)	Unable to speak or understand Dutch
Mentally competent as measured by judgment of the GP **OR** if Mini-Mental State Examination has been conducted, score is > 24	Unable to provide consent or complete the questionnaires due to cognitive impairment (as judged by the GP)
GP answers “no” to surprise question: “Would I be surprised if this patient were to die within the next 12 to 24 months?”	GP answers “no” to surprise question: “Would I be surprised if this patient were to die within the next 6 months?”
Diagnosis of a life-limiting illness: 1. Locally-advanced unresectable, or metastasized **cancer** OR 2. **Organ failure,** this being a) heart failure (New York Heart Association stage 3 or stage 4) b) chronic kidney failure or end-stage renal disease (ESRD) (stage 4, eGFR = 15–29; or stage 5, eGFR< 15) c) Very severe COPD (GOLD COPD stages stage 3 or stage 4) OR 3. **Geriatric frailty** (Clinical Frailty Scale score 5–7, mildly to severely frail)	Participated in the pilot study of this intervention or in the cognitive testing of the adjusted intervention materials
	Participating in other studies evaluating advance care planning, palliative care services or communication strategies
**SDM inclusion criteria**	**SDM exclusion criteria**
Adults (> 18 years old)	Unable to speak or understand Dutch
Identified by the patient as their surrogate decision maker OR as a person who may be willing to be their surrogate decision maker	Unable to provide informed consent

The patient may identify their SDM for inclusion, or they may designate someone who may be willing to act as their SDM; the latter is the potential SDM. Through the rest of this manuscript, “SDM” will refer to both the SDM and the potential SDM. While patients are encouraged to identify a SDM for participation, not identifying one will not exclude the patient from participation.

All inclusion criteria for patients and their SDM can be found in Table 1.

### Intervention and control

#### Intervention

The ACP-GP intervention (Table 2) is designed to 1) train GPs to conduct ACP discussions with eligible patients, 2) prepare patients for the conversation by providing them with a workbook about ACP, 3) facilitate at least 2 ACP conversations between GP and patient (and SDM if present), and 4) document the outcomes of the discussion in the patient electronic medical file with the help of a structured template.

**Table 2:** Key elements of the ACP-GP intervention

1. GP training	The GP training, which has been tested multiple times, is originally conceptualized as two interactive sessions of 3 h each, delivered to small groups of 6–8 GPs at a time within the university hospital setting or another location that is convenient for the participants. However, due to COVID-19 concerns, the content of the training has been translated to an online platform. The training is provided by a trainer experienced in primary care and communication. Two interactive web sessions of approximately 2 h each will replace the live sessions. Preparatory activities such as fictional case examples with reflection questions will be available before the training begins. GPs will have access to background information portions through an e-learning module presented via the Ufora platform of the Universiteit Gent. This module will take no more than 60 min to review. The first aim, improving ACP knowledge, will be addressed via the e-learning module. ACP communication skills will be practiced with video role-modeling exercises which are available on the e-learning module and will be further elaborated on during the web sessions. These web sessions will also include role-play exercises with model patients and interactive discussions with fellow GPs and the trainer. During the training, GPs will receive an extensive conversation guide and an at-a-glance conversation flowchart. These can be used as preparation for and during ACP conversations with patients. In the context of their continuous medical education, GPs will be able to obtain accreditation in ethics and economy by following the training. GPs in the control group will have the opportunity to follow the training after the conclusion of the study, so that both groups have access to this incentive. After the training sessions, GPs will have the opportunity for check-in discussions with the trainers to ask questions and report issues.
2. ACP workbook for patients	During the first home visit, the RA will give patients an ACP workbook that highlights the importance of ACP at different stages of health. The workbook contains questions to stimulate reflection on topics such as quality of life and preferences for future care. The workbook is adjusted for health literacy and has been evaluated through cognitive interviewing with 6 patients who fulfill the inclusion criteria of the trial.
3. Patient-centered ACP discussion with conversation guide	After the training, the GP will conduct a minimum of two ACP conversations in the patient’s home or in the GP office. If COVID-19 safety concerns prohibit the GP from speaking face-to-face with the patient, a telephone consultation or video-consult via an accredited electronic health record software package is also possible. The first conversation takes place within 2 weeks after the GP has received the training; the second within a month after the first conversation. The GP can use the conversation guide, which contains parallel topics to the patient workbook, to structure the conversation. First, the patient is invited to talk about the questions and topics they saw as most important. Then, if time permits, the conversation moves to the questions that have not yet been discussed Patients can choose to have their SDM present at these conversations. If the patient has not yet identified an SDM, they will be encouraged to think about who might be a good fit for this role. Other already-available documents, such as advance directive forms or patient guide materials such as the information booklet provided by the LevensEinde Informatie Forum (LEIF), may be used as the GP or patients see fit. The ACP discussion is expected to last up to 60 min, but GPs are advised during the training to judge the optimal duration according to the openness and engagement of the patient.
4. Documentation of the ACP discussion	The GP will fill out a template reflecting the outcomes of each ACP conversation. The template is based on the structure of the conversation guide. Here, the GP can freely note what was discussed, even if no concrete care decisions were made. During the training, the GP will be instructed to upload this documentation to the patient’s electronic medical file. With the patient’s permission, this information can be shared with other health providers involved in the patient’s care, such as specialist practitioners and home care nurses

#### Control: care as usual

The intervention will be compared to a control group, which is care as usual. In this group, participating GPs will not receive the training or the conversation guides, and patients will not receive the workbook developed for the intervention. The control group will also not feature the two planned consultations dedicated specifically to discussing ACP as included in the intervention arm. Participating patients will consult with their GP as they usually do. During these consultations, the topic of ACP may still spontaneously be addressed, either by the GP or patient. Other already-available national documents, such as advance directive forms or patient guide materials, may be used as the GP or patients see fit.

#### Criteria for discontinuing or modifying allocated interventions for a given trial participant

Participants may discontinue their participation at any time and for any reason, as is described in the informed consent forms. Patients will be monitored by the researchers for the possibility of adverse events and may discontinue their participation in response to adverse events, the detection of which will prompt a notification of the trial manager and the ethics committee.

#### Strategies to improve adherence to intervention protocols, and any procedures for monitoring adherence

Trainers who provide the intervention training to GPs will be trained by researchers who developed the training and provided the training during the pilot study. One of the trainers, PP, is an instructor to GPs-in-training who also conducts training sessions on the topic of palliative care. Therefore, the train-the-trainer model is based on the expertise and experience of the primary trainer, improving the quality of the training.

The regular check-in moments with the GPs by the trainers as part of the process evaluation will also serve as a means to monitor adherence to the study protocol. During the check-in moments, GPs will be asked to report on how they are delivering the intervention and which problems they are encountering. This allows the trainers to detect difficulties the GP might have in delivering the intervention or take note of a possible lack of intervention fidelity. If necessary, the trainers can remind the GP of the study protocol and/or answer questions the GP might still have.

Verbatim transcriptions of audio-recorded ACP conversations as well as anonymised completed ACP documentation templates will be used to evaluate fidelity and adherence to the study protocol. Additionally, at T1 we will provide GPs in both groups with a process evaluation questionnaire which asks them to report the number of ACP conversations conducted with each participant, the length of each conversation, the topics discussed, where the conversation was documented, and who was present during the conversation.

A sample of completed, anonymised workbooks from patients will be examined to check to what extent the workbook is used, which questions are more or less frequently answered, and whether patients document having discussed the workbook with others (for which a simple table is provided on the final workbook page; this can include the SDM but can also be other family members, health providers, friends, etc.).

### Relevant concomitant care and interventions that are permitted or prohibited during the trial

There are no restrictions regarding concomitant medical care or medical interventions during the trial period. Participants may receive care as normal, with the exception of participation in other studies or trials evaluating ACP interventions, palliative care services, or other communication strategies. Patients participating in such studies or trials will be excluded from participating in this study.

### Outcomes

#### Study endpoints and assessments

This study uses both qualitative and quantitative data to measure the outcomes of the intervention. As the intervention consists of components developed for the GP and patient, outcomes will be measured at both levels. The primary and secondary outcomes are listed in Table 3.

**Table 3:** Outcomes, measurement instruments and timing

	Measurement tool	Completed by	Timing of measurement		
Primary outcome			T0	T1	T2
Level of engagement with ACP	*ACP Engagement Survey 15-item version* [24] • Reported on an overall average 5-point Likert scale (range 1–5)	Patient	X	**X**	X
ACP Self-efficacy	*ACP Self-efficacy Scale (ACP-SE)* [25] • 17 items • Reported on an overall average 5-point Likert scale (range 1–5) • 1 additional general item including all advance care planning can be used for comparison to the scale	GP	X	**X**	X
Secondary outcomes					
Health-related quality of life	*Short Form Health Questionnaire (SF-12v2)* [26] • Physical Health (PCS) and Mental Health (MCS) summary scores (range 0–100)	Patient	X	X	X
Anxiety	*Generalized Anxiety Disorder Questionnaire (GAD-7)* [27] • Sum score (range 0–21)	Patient	X	X	X
Depression	*Patient Health Questionnaire (PHQ-9)* [28] • Sum score (range 0–27)	Patient	X	X	X
Appointment of a substitute decision maker	GP report ACP engagement survey “readiness to sign official papers assigning a SDM” item	Patient GP	X	X	X
Completion of new ACP documents	Patient report GP report ACP engagement survey “readiness to sign official papers stating medical wishes” item	Patient		X	X
Thinking about ACP	1 self-developed item, 10-point Likert (“How much have you thought about ACP in the last 3 months?”; response categories range from “not at all” to “very much”)	Patient	X	X	X
Communication with the GP	4 self-developed items, 10-point Likert (e.g., “To what extent did the GP listen to your concerns about your future health?”; response categories range from “not at all” to “very much”)	Patient	X	X	X
ACP Practices	• *Next Steps training program questionnaire* [29] (4 items) • 2 items specific to practices with patients with chronic, life-limiting illness (“Which percentage of your patients has a chronic, life-limiting illness” and “With which percentage of your patients with a chronic, life-limiting illness do you conduct ACP conversations?”; 4 response options per item) [25] • 8 additional items regarding ACP practices (e.g., “Where do the ACP conversations you conduct usually take place?”)	GP	X	X	X
ACP Attitudes	*Next Steps training program questionnaire* [29] • 9 items; 5-point Likert scale ranging from “Completely disagree” to “Completely agree”; adapted to the Belgian legal context	GP	X	X	X
ACP Knowledge	*Next Steps training program questionnaire* [29, 30] • 10 items; correct/not correct/don’t know; adapted to the Belgian legal context	GP	X	X	X
Documentation of ACP discussion outcomes	Documentation template review	GP		X	X
Level of engagement with ACP	*ACP Engagement Survey, substitute decision maker version* [31] • 17 items; 5-point Likert scales • 3 domain scores (“Serving as SDM”, “Contemplation”, Readiness”) computed as the unweighted average of items per domain (range 1–5)	SDM	X	X	X
Other measurements					
Demographic information	For patients and surrogate decision makers: • Gender • Age • Marital status • Highest completed education • Religion • Patient/SDM relationship • Whether patient and SDM live together or apart For patients: • Previous completion of any advance directives (“wilsverklaringen”) For surrogate decision makers: • How long they have known the patient For GPs: • Gender • Age • Graduation year • Practice setting(s) • Years of experience as a GP • Graduating university • Working in a palliative home care team (yes/no) • Working as a “coordinating and advising practitioner” in a residential care facility (yes/no) • Prior formal ACP education or training (intensive/introductory/none) • Prior formal palliative care education or training (intensive/introductory/none)	GP Patient SDM	X		
Process evaluation					
**RE-AIM domain**	**Operationalization**	**Measurement**			
**Reach**	• Comparing the characteristics of participating patients with non-participants	• Documentation of the recruitment process by the researchers • Documentation of reasons given for not participating • Participant demographics			
**Efficacy/effectiveness**	• Primary and secondary outcomes of the RCT	• See primary and secondary outcomes above • Reports of any adverse effects			
**Adoption**	• ACP discussion documents uploaded • Patient use of the work booklet • Experiences of GPs and patients applying intervention steps • Changes in GP practice	• Training topic checklist (after each training) • Questionnaire for GPs regarding their ACP practices and conversations in the last 3 months (T1) • Questionnaire for patients regarding ACP conversations with their GP in the last 3 months (T1) • Documentation template review (T1, T2) • Contents of work booklet from a sample of patients in the intervention group (physical copy or digital scan) (T1, T2) • Check-in discussions between GPs and trainers (continuous) • Focus groups with GPs (after T2) • Semi-structured interviews with patients and SDM (after T2)			
**Implementation**	• Fidelity: the extent to which the steps of the intervention were followed as specified in the protocol • Patient and GP barriers/facilitators to following the steps of the intervention • Satisfaction of GPs and patients with the intervention components	• Training topic checklist (after each training) • Check-in discussions between GP and trainers (continuous) • Audio recordings of ACP consultations between GP and patient (and SDM if present) • Documentation template review (T1, T2) • Satisfaction questionnaire for intervention GPs and patients (T1) • Focus groups with GPs (after T2) • Semi-structured interviews with patients and SDM (after T2)			
**Maintenance**	• GP intention for using the intervention materials in the future • Recommendations by the GP and patients to improve intervention usability in the future	• Satisfaction questionnaires for intervention GPs and patients (T1) • Focus groups with GPs (after T2) • Semi-structured interviews with patients and SDM (after T2)			

We have two separate primary outcomes, one at the GP level and one at the patient level. Success on any one of these outcomes at T1 may support a conclusion of effectiveness. Hence there are several ways for the study to successfully demonstrate a treatment effect. This multiplicity problem has been taken into account in the power analysis by controlling the Type I error rate at 2.5% (Bonferroni method).

Scores on the ACP Engagement Survey and the ACP Self-Efficacy Scale for GPs will also be measured at T2. We will treat T2 scores on these scales as a secondary outcome.

#### Process evaluation

A process evaluation will be used to evaluate how the intervention was implemented and to understand which factors contributed to the results of the trial. This process evaluation follows the RE-AIM framework, which highlights essential factors to improving the adoption and implementation of evidence-based interventions: Reach, Efficacy, Adoption, Implementation, and Maintenance [20]. The process evaluation will span the duration of the intervention, as well as pre and post-intervention evaluation. An overview of the process evaluation, with RE-AIM domains and data collection methods, can be found in Table 3.

### Participant timeline

**Fig. 1:**
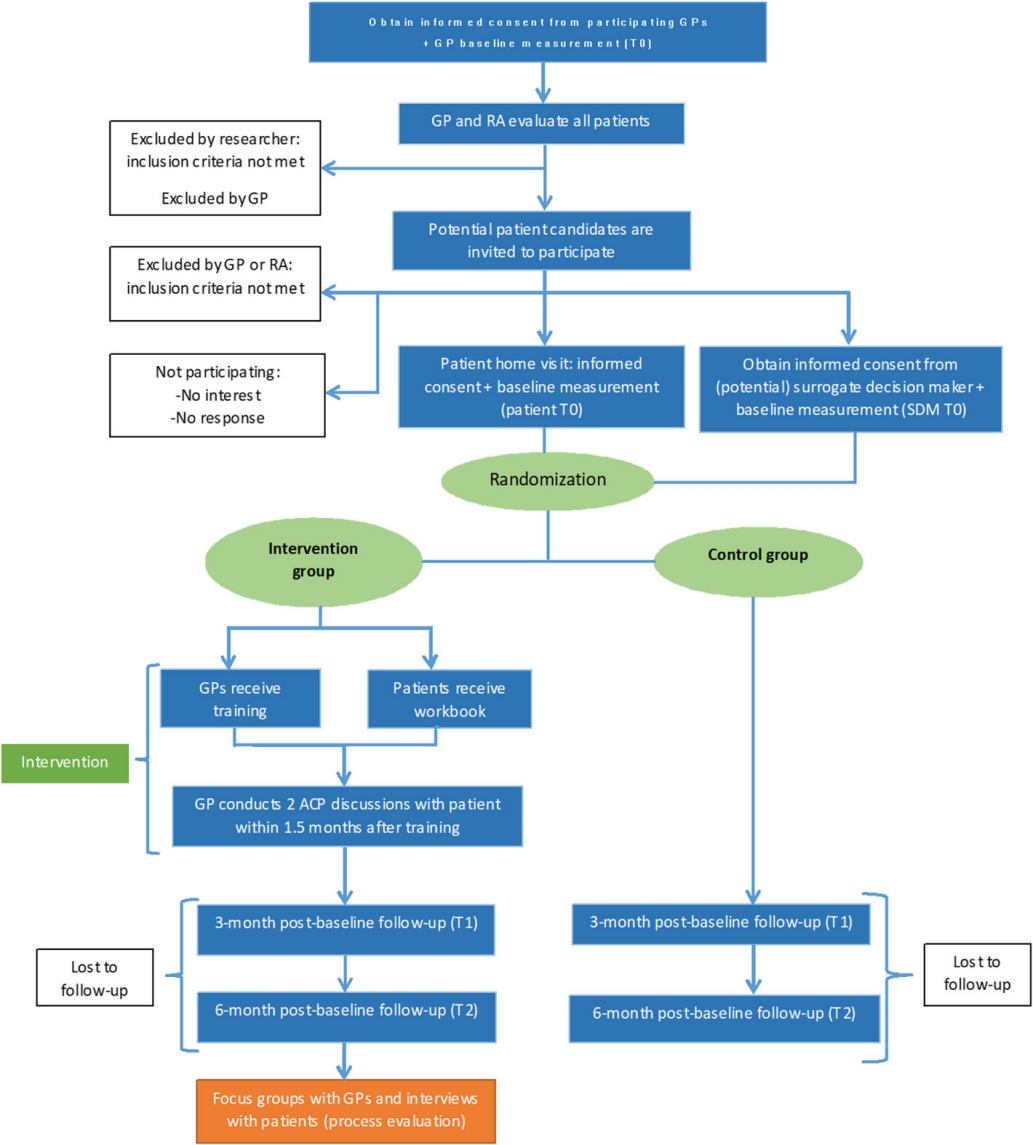
Flow diagram of the ACP-GP trial

The participant timeline flow diagram is represented in Fig. 1. All GPs, patients, and SDMs from the intervention and control group will complete a baseline assessment (T0) after providing informed consent. At 3 months (T1), the RA will approach the patient and SDM for follow-up assessment; GPs will complete a follow-up assessment by filling out and returning questionnaires by postal mail or by completing an online version of the questionnaire. Six months (T2) after inclusion, patients and SDMs will complete the second follow-up by filling out and returning questionnaires by postal mail; GPs will also complete follow-up measures at this time by filling out and returning questionnaires by postal mail or by completing an online version of the questionnaires.

### Sample size

All power calculations were conducted to allow testing for intervention effectiveness at T1. Power calculations were conducted for the primary outcome at the patient level and at the GP level.

When all clusters have the same size of 2 patients, and we assume an intracluster correlation coefficient (ICC) of 0.04 [32], then the design effect is estimated at 1.04, and a sample of 26 patients for each group (corresponding to 13 GPs with each 2 patients) will achieve 91.45% power to detect a mean difference in delta outcome of 1 at a significance level of 2.5%, assuming the standard deviation is 0.96 in both groups. This number has been increased to 51 patients per group (corresponding to 17 GPs with each 3 patients) to allow for an initial GP drop-out rate of 23.53% and a patient drop-out rate of 33.33%. (Total sample size of 102 patients).

A sample of 14 GPs for each group will achieve 91.11% power to detect a mean difference in ACP self-efficacy of 1 at a significance level of 2.5%, assuming the standard deviation is 0.71 in both groups. This number has been increased to 18 GPs per group to allow for an initial GP drop-out rate of 22.22% (Total sample size of 36 GPs).

To ensure sufficient statistical power for both GP- and patient-level primary outcomes, we will use the more conservative calculation of 18 GPs per group (36 total), with 54 patients per group (108 total) and a maximum of 54 SDMs per group (1 per patient, 108 total).

### Recruitment

#### GPs

The research team will recruit GPs through several channels. Quality peer-review groups will be contacted to provide information about the study and motivate participation to their members. Publicly-available member lists of local associations of GPs and contact lists of GPs will be used for telephone and email contact and for providing recruitment letters by postal mail.

GPs who are eligible and wish to participate will be asked to provide informed consent. Each GP will be asked to list, with the help of a research assistant (RA), three patients who are potentially eligible for participation in the study. Where possible, each of the three identified patients should have a different life-limiting illness (cancer, organ failure, geriatric frailty) according to the criteria listed in Table 1.

#### Patients

GPs will have approximately 1 month to present the study to their selected eligible patients and ask them if they wish to participate. A simplified information letter will be provided to help explain the study. If the patient does not wish to participate, the GP will be asked to identify another potentially eligible patient within the same category of life-limiting illness. Eligible patients who agree to participate will be contacted via telephone by the research team, who will provide information about the study during a visit at the patient’s home or other location that is convenient for the patient, or via telephone if COVID-19 safety concerns prohibit face-to-face contact.

If a GP drops out during the first 3 months of the trial, a new GP will be recruited through the channels described below and trained to use the intervention. Reasons for drop-out will be discussed, recorded and taken into account for the process evaluation. If a GP withdraws from the study or drops out after 3 months, no new GP will be recruited. As only 3 patients will be enrolled per GP and the sample size has been increased to allow for a 23.53% GP drop-out and 33.33% patient drop-out, the drop-out of any one practice will not greatly impact the study.

#### SDMs

RA’s will assist patients with identifying a SDM using a pre-written script which asks whether the patient has formally appointed someone, and if not, who may be able or willing to fulfill this role. If the SDM is present at the time of the visit, they will be asked if they would like to participate. If the SDM is not present, the researchers will ask permission from the patient to contact them regarding the study. If such a person wishes to participate, they will also be asked to provide informed consent.

### Assignment of interventions

#### Allocation

Randomization will be performed at the level of the GP to avoid contamination bias. Every GP will have to recruit at least three patients. Once three patients have been recruited, the GP will be randomized to either the intervention or control group according to a 1:1 allocation ratio per a computer-generated randomization list. We will use permuted block randomization with varying block sizes. No stratification factors will be taken into account.

Participants will be enrolled to the study by the research assistants and data managers. The allocation sequence will be generated by independent statisticians working with the Biostatistics Unit at the Faculty of Medicine and Health Sciences of Ghent University. Assignment to intervention or control groups of GP-patient clusters will be performed by a researcher not involved with any other portion of this study.

#### Blinding

Due to the nature of the intervention, neither GP, patient, nor SDM participants can be blinded to allocation. Although the participants cannot be blinded to their assignment and researchers will be unblinded to GP assignment through the coordination of the training sessions, data collection at T0, T1, and T2 will be performed by an independent data collector/research assistant who is blinded to the assignment of the GP and patient to either the intervention or control arm. Those performing the data analysis will likewise remain blind to participant allocation.

Informed consent and data collection at T0 will occur before randomization has taken place.

### Data collection, management, and analysis

#### Data collection methods

The outcome measures and general procedures for data collection are described above. Questionnaire data will be collected at T0, T1, and T2. GPs have the option to complete the questionnaires on paper or online if preferred. Patients and SDMs will complete questionnaires on paper. Patients and SDMs completing the questionnaires at T0 and T1 can receive support from a data manager if so desired, either through an in-person visit by the data manager or via telephone contact. If COVID-19 safety concerns arise which prohibit home visits, all support will be provided through telephone contact.

As described above, data collection for the process evaluation will occur during and after the intervention period by means of questionnaires, documentation of activities, and audio-recordings of ACP conversations. These audio-recordings will be transcribed for analysis. During the recruitment phase, those approached for recruitment who do not wish to participate may optionally provide their reason for not participating.

After the 6-month intervention period has elapsed, the process evaluation will be continued through interviews and focus groups with patients and their SDMs, and GPs respectively. With permission from the participants, focus groups and interviews will be audiotaped to allow for later verbatim transcription. The interviewer will also take notes during the interviews and focus groups. Both the interviews and the focus groups will be conducted according to a topic list, with attached instructions for the interviewer (or moderator and observer for focus groups).

With patients in the intervention group and their SDM, if one was identified, 10–15 interviews will be conducted. Focus groups with GPs will include 6–8 GPs per focus group. Interviews and focus groups will be conducted until data saturation is achieved; that is, until the newly-collected data is redundant with the already-collected data and no new results emerge.

To improve retention, participants will be presented with a gift certificate for their participation in the study. Additionally, the consultation costs for the first two ACP discussions planned in the intervention group (i.e., the consultations required for the intervention) will be compensated by the researchers.

#### Data management

To pseudonymize the data, each participant will be assigned a study identification number. A list with identification codes which links the participant’s name to the participant’s identification number will be stored in a limited-access space. Response input will only use the participant identification number. All digitally inputted data will be stored on a secure server. Access to this server is strictly limited to those who require access to conduct the study. All informed consent forms will be stored in a lockable filing cabinet restricted to members of the research team. Paper questionnaires will be stored in a separate lockable filing cabinet with similar restrictions.

Data will be retained for 10 years, after which it will be destroyed. Data will be shared only for the purposes of the study and will not be shared with other countries.

A trial manager will take responsibility for the data management over the course of the study. A record of the study and its data processing activities has been submitted to the Data Protection Office (DPO) of the Vrije Universiteit Brussel.

#### Data analysis

The intent-to-treat population consists of all patients randomized. Subjects are analyzed according to the allocated treatment group irrespective of their compliance with the planned course of treatment. The intent-to-treat population is considered the main analysis population.

Linear mixed models will give unbiased results when outcome data is missing at random (maximum likelihood estimation). GEE models only allow for missing values to be completely at random (it is not a likelihood approach).

The analyses of the two separate primary endpoints will be performed at the two-sided 2.5% significance level, because success on any one of these endpoints at T1 may support a conclusion of effectiveness (Bonferroni method to adjust for multiplicity).

When an effect on a primary endpoint is shown, the secondary endpoints can be analyzed at the two-sided 5% significance level.

##### Descriptive statistics

Demographic characteristics of participants (at T0) will be summarized using descriptive statistics (absolute and relative frequencies for nominal variables, mean and standard deviation for continuous variables with normal distribution, median and 25-75th percentiles for continuous variables without normal distribution).

##### Primary efficacy analyses

To test the effectiveness of the intervention, we will compare T1 scores on the ACP Engagement Survey for patients and the ACP-Self Efficacy Scale for GPs between the intervention and control arms. Linear mixed models will be used. For patient outcomes, the models will include a random intercept for GP (to account for the nesting of patients within a GP) and a random intercept for patient (to account for the nesting of repeated measurements within a patient). For GP outcomes, a random intercept for GP (to account for the nesting of repeated measurements within a GP) will be used. The fixed effects part of these linear mixed models will include time, group, and time x group interaction. The two-way interaction between time and group will capture the effect of the intervention.

##### Secondary efficacy analyses

All continuous secondary endpoints will be analyzed by fitting similar linear mixed models as described above. Binary, multinomial, ordinal and count endpoints will be analyzed by fitting Generalizing Estimating Equations (GEE) models using a compound symmetry (or exchangeable) correlation structure, where we assume all correlations between time points to be the same. The GEE approach is a robust approach to take into account the repeated measurements within GPs without distributional assumptions. It is robust against misspecification of the covariance structure. However, it only allows missing values to be missing completely at random. Only an independent correlation structure is available for multinomial GEE models in SAS and SPPS software. Therefore, for nominal endpoints with K response categories, we will fit K-1 separate binary logistic GEE models for each response category paired with a baseline category.

##### Process evaluation

Process evaluation of the implementation of the intervention will be analysed following the RE-AIM framework. For the process evaluation, questionnaires will be analysed as follows:

Standard tests for independent data will be used to compare the questionnaires regarding ACP conversations in the last 3 months, completed by patients and GPs. Comparisons will be per item. For patients, clustering within GPs will be taken into account.

Descriptive statistics will be used to summarize the responses for the satisfaction questionnaires completed by patients and GPs. Absolute and relative frequencies of response options will be reported.

We will calculate descriptive statistics for any additional quantitative measures such as recruitment documentation, checklists of the training topics, and use of the workbook and documentation template. For document reviews such as that of the workbook, the process evaluation will only consider which items were answered, not the content of the answers.

Transcribed recordings, as well as interviewer notes from the focus groups with GPs and interviews with patients and SDMs, will provide the qualitative data for the process evaluation. Transcriptions and notes will be analysed line-by-line using NVivo. The comments and feedback given during the focus groups/interviews will be analysed via coding, combining and clustering based on common themes, and subcategorizing based on item interpretation. Using these codes, the research team will identify dominant response trends. During team discussions, the findings, interpretations, and conclusions across items will be reviewed in order to reach a consensus regarding potential problems with the materials. During these discussions, possible resolutions will be suggested. The qualitative analyses of the transcribed recordings will be carried out by JS as well as by research team members ADV, KP, KE, and LD.

### Data monitoring

#### Data monitoring

This study will not have a Data Monitoring Committee. Excel sheets will be used to monitor recruitment and study responses. The research team will meet regularly (bi-weekly to weekly) during the recruitment period to review recruitment.

In the case of nonresponse to questionnaires, a follow-up notice will be sent to participants: GPs will receive a notice by mail and email, and patients and SDMs will receive a notice by mail. If there is no response after this notice, a final telephone follow-up will be conducted. Questionnaire forms, in Dutch, are available from the authors upon request.

##### Interim analyses and stopping guidelines

Analysis of data will begin when baseline data has been collected for all participants, to compare participant demographics and evaluate reasons for refusal to participate. Data will be analyzed for primary endpoints at T1 (3 months post-baseline). If the trial must be terminated at any point before the completion of both qualitative and quantitative outcomes as described in Table 3, this will first be discussed with the researchers during an internal meeting. The final decision to terminate the trial can be made by Prof. Dr. Koen Pardon or Prof. Dr. Luc Deliens after this meeting. Should the trial be terminated this will be reported to the ethics committee, the data protection office, and the funder.

#### Harms

The ACP intervention is a non-invasive intervention, focused on conversations between GPs, patients, and SDMs regarding values and wishes for future medical care. Previous research has shown that people with life-limiting illnesses see participating in research such as this study as a worthwhile endeavor [17]. Adverse effect from participating in similar research, as may be implied by dropout due to the subject being too psychologically taxing to talk about, are rare [33]. However, people living with chronic life-limiting illnesses are a vulnerable group for whom the appropriate concern and ethical measures must be in place. An anticipated adverse event which may arise during the intervention is mild psychological discomfort in participating patients and SDMs, which may be caused by some questions in the ACP Engagement Survey or the workbook, or as a result of ACP conversations with the GP. However, participants will be informed of their right to refuse to answer any question and that they may withdraw from the study at any time without negative consequence. The possibility of a patient or SDM becoming distressed during the ACP discussions will be discussed during the GP training.

As the study involves patients with a chronic, life-limiting illness, it is possible that some patient drop-out is due to death related to disease progression, but this would not be related to the study protocol. A bereavement protocol has been established for the SDM if the patient dies during the study period. If researchers are informed that a patient has died, this will be communicated to the ethics committee. The bereaved SDM will be contacted with condolences and, if necessary, will receive information to refer them to appropriate support resources.

While the investigators cannot predict the occurrence of unanticipated or unexpected adverse events, we do not anticipate any serious adverse events associated with the research protocol. Nevertheless, we have included measures to detect increases in anxiety and depression at T1 and T2 and will act accordingly in the case of adverse events. These will be reported to the principal investigator and forwarded to the ethics committee. In the case of an adverse event involving a patient, the GP and specialist health provider to the patient will be notified if necessary.

Any adverse event will be reported to the Medical Ethics Committee (Commissie Medische Ethiek) of the VUB. If the adverse event is associated with the study, an internal discussion with the research team will be conducted alongside a consult with the ethics committee regarding the need to revise the study procedures, to prevent a recurrence of similar adverse events.

### Ethics and dissemination

#### Confidentiality

All collection and processing of personal data will proceed in compliance with EU Regulation 2016/679, General Data Protection Regulation (GDPR) (Europese Algemene Verordening omtrent Gegevensbescherming (AVG)). Participants will be informed of their rights to confidentiality under this regulation according to a standardized text provided by the Medical Ethics Committee.

Questionnaires completed online will ask participants to enter their personal participation code in order to proceed. Questionnaires completed on paper will also use this personal participation code written in the header of the questionnaire form. Participants will not be asked to enter their names. Transcriptions of audio recordings will pseudonymize any names of persons. In no case will video recordings be made of participants.

#### Ancillary and post-trial care

In the case of an adverse event for a patient during the study, the GP and, if necessary, the specialist health provider to the patient will be notified to further refer the patient to existing medical care services. The researchers will also be available to refer patients and SDMs to appropriate supportive resources based on needs identified during the study.

#### Dissemination policy

At least four articles are planned based on the results of this study: 1. Baseline findings, 2. Patient and SDM outcomes, 3. GP outcomes, and 4. Qualitative and process evaluation outcomes. These articles will be written within the scope of a PhD dissertation. Furthermore, the study findings will be communicated through contributions to (inter) national conferences in the fields of advance care planning and end of life care. On a national level, we will collaborate with general practice organizations and disseminate the results of the study through professional journals of key stakeholders in Belgium. Once evaluated, the training component of the intervention can be incorporated into teaching activities for students, researchers, and healthcare professionals. The patient workbook can similarly be updated for uptake in GP and other health settings.

## Discussion

The aims of this project focus on facilitating ACP in general practice, where great improvements can be made towards timely and recurring communication about care preferences with patients with chronic, life-limiting illness [8].

The ACP-GP intervention utilizes the unique position of GP’s and their relationship with their patients. By providing GPs an opportunity to increase their ACP knowledge and communication skills through an interactive training, GPs may feel more prepared and confident to initiate these conversations [34]. For patients, a workbook that encourages reflection and discussion about questions essential to ACP can more adequately prepare them for ACP conversations with the GP. By tailoring ACP conversations to the patient’s readiness, health behavior change is facilitated over the course of recurrent discussions. Therefore, changes in behavior change states, even in the absence of action outcomes such as AD documentation in the short term, can be indicative of an ACP process [35, 36].

This trial will be the first study in Belgium to conduct a large-scale evaluation of the impact of an ACP intervention in general practice on patients’ level of engagement with ACP. The intervention has a strong theoretical basis, developed through literature research and stakeholder participation at every point in the process [15, 16], following the recommendations of the MRC framework. The addition of a process evaluation using the RE-AIM framework allows us to identify specific barriers and facilitators to the successful implementation of the intervention. By measuring at 3 and 6 months post-inclusion, we will also be able to show the sustainability of the intervention in the long term, which is important when including patients with chronic, life-limiting illness who are however not yet close to death.

Some challenges can be anticipated. First, the pilot study of this intervention showed that a perceived lack of time to undertake ACP discussions may prevent some GPs from participating [17, 37]. ACP conversations intrinsically will require a certain time investment from GPs. Preparing patients for these conversations using the workbook and training GPs in ACP communication may allow for more efficient use of this time, and can save time when the patient is nearing the end of life and treatment decisions must be made. The researchers have also made efforts to limit the time investment, for example by supporting the GP during the identification of eligible patients. Second, asking GPs to designate patients for inclusion may introduce a selection bias towards patients with whom the GP feels comfortable discussing ACP. This decision was the result of extensive deliberation within the research team, which includes a GP. We consider it inappropriate to interfere in existing GP-patient relationships by imposing ACP conversations on patients who are not at all open to, or would be extremely distressed by, such conversations. Third, data collection at three time points using questionnaires may burden patients and increase the risk of nonresponse. To address this, data collectors will be present during T0 and T1 data collection, and will conduct telephone follow-up for T2 questionnaires. Fourth, blinding of participants is not possible during the study period as GPs will receive additional training and patients will receive the workbook and additional consultations for ACP conversations. A lack of blinding may affect the answers of patients or GPs who are aware of their group assignment. This limitation frequently occurs in ACP intervention studies, where many past trials have also been unable to blind participants [38, 39]. Finally, questionnaires administered to the control group may raise patient and SDM awareness of ACP, potentially increasing their engagement. However, ACP resources which are already generally available can be accessed by both groups and both groups will complete the ACP Engagement Survey. If the intervention is effective, we expect to find differences between the intervention and control group even when these assessment effects are taken into account.

## Conclusion

General practitioners play a critical role in the timely initiation of ACP, but barriers remain and little evidence exists of how GPs and patients can effectively prepare for and engage in these conversations. The ACP-GP intervention study will provide valuable evidence for the implementation of ACP in general practice and for the effectiveness of tools developed to facilitate these conversations.

## Supplementary Information

**Additional file 1.** SPIRIT 2013 Checklist. Recommended items to address in a clinical trial protocol and related documents.

## Data Availability

Not applicable.

## Abbreviations

ACP: Advance Care Planning
ACP-GP: Advance Care Planning intervention for General Practice
ACP-SE: ACP Self-Efficacy Scale
AD: Advance Directive
GAD-7: Generalized Anxiety Disorder-7 Questionnaire
GEE: Generalizing Estimating Eqs.
GP: General Practitioner
ICC: Intra-Cluster Correlation Coefficient
MRC: Medical Research Council
PHQ-9: Patient Health Questionnaire-9
SDM: Surrogate Decision Maker
SF-12v2: Short Form Health Questionnaire-12 version 2
SPIRIT: Standard Protocol Items: Recommendations for Interventional Trials
RCT: Randomized Controlled Trial
RE-AIM: Reach, Efficacy, Adoption, Implementation, Maintenance
